# A resurrection study reveals limited evolution of phenology in response to recent climate change across the geographic range of the scarlet monkeyflower

**DOI:** 10.1002/ece3.7011

**Published:** 2020-11-13

**Authors:** Emma E. Vtipil, Seema Nayan Sheth

**Affiliations:** ^1^ Department of Plant and Microbial Biology North Carolina State University Raleigh NC USA

**Keywords:** drought escape, *Erythranthe*, evolutionary rescue, flowering time, geographic range, *Mimulus*

## Abstract

**Premise of the study:**

As global climate change alters drought regimes, rapid evolution of traits that facilitate adaptation to drought can rescue populations in decline. The evolution of phenological advancement can allow plant populations to escape drought, but evolutionary responses in phenology can vary across a species' range due to differences in drought intensity and standing genetic variation.

**Methods:**

*Mimulus cardinalis*, a perennial herb spanning a broad climatic gradient, recently experienced a period of record drought. Here, we used a resurrection study comparing flowering time and stem height at first flower of pre‐drought ancestors and post‐drought descendants from northern‐edge, central, and southern‐edge populations in a common environment to examine the evolution of drought escape across the latitudinal range.

**Key results:**

Contrary to the hypothesis of the evolution of advanced phenology in response to recent drought, flowering time did not advance between ancestors and descendants in any population, though storage condition and maternal effects could have impacted these results. Stem height was positively correlated with flowering time, such that plants that flowered earlier were shorter at first flower. This correlation could constrain the evolution of earlier flowering time if selection favors flowering early at a large size.

**Conclusions:**

These findings suggest that rapid evolution of phenology will not rescue these populations from recent climate change. Future work is needed to examine the potential for the evolution of alternative drought strategies and phenotypic plasticity to buffer *M. cardinalis* populations from changing climate.

## Graphical abstract

We evaluated whether populations across the geographic range of the scarlet monkeyflower have evolved earlier flowering times in response to a recent period of severe drought and warming. We hypothesized that populations would vary in the magnitude of their evolutionary responses, depending on standing genetic variation and the magnitude of recent drought. None of the populations evolved earlier flowering times, suggesting that they may need to rely on other means such as range shifts, phenotypic plasticity, or evolution of drought avoidance to cope with increased drought.

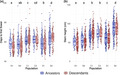

## 1 INTRODUCTION

Global climate change, including rising temperatures and increases in the frequency and severity of drought (Briffa et al., 2009), is shifting where species occur and how they evolve (Hamann et al., 2018; Parmesan & Yohe, 2003). Species can persist in the face of climate change via range shifts (Parmesan & Yohe, 2003), phenotypic plasticity, and evolutionary adaptation (Williams et al., 2008). When plastic changes are limited, adaptation is crucial to avoid extinction, and can occur in situ or synchronously with range shifts (Davis & Shaw, 2001). In the face of environmental change, population persistence may depend on evolutionary rescue. Evolutionary rescue is characterized by a depression in population abundance due to environmental change, followed by adaptive evolution restoring the population above replacement levels (Carlson et al., 2014). The probability of evolutionary rescue, in part, depends on standing genetic variation in ecologically important traits, as well as the rate of environmental change, and recent work suggests that evolutionary rescue is most likely under high genetic variation and gradual environmental change (Carlson et al., 2014). Rapid evolution in response to recent climate change has been documented across multiple taxa (Anderson et al., 2012; Franks et al., 2007; Lustenhouwer et al., 2018), suggesting that populations can harbor sufficient genetic variation to respond to climate‐induced selection. Yet, populations may vary in their abilities to adapt to changing climate, and whether evolutionary rescue can occur in the face of rapid environmental change remains unknown.

Populations across a species' range can vary in their abilities to rapidly evolve under climate change due to spatial variation in selection and genetic variation in ecologically important traits. First, the magnitude of climate change, and thus presumably the strength of selection on traits involved in adaptation to climate, varies across space (Swain et al., 2018). For instance, higher latitudes are experiencing greater warming (IPCC, 2013), and drought intensity differs among geographic regions (Clark et al., 2016). Similarly, the strength of selection can vary across latitudinal gradients, leading to local adaptation (Etterson, 2004). Second, populations may harbor different levels of genetic variation in ecologically important traits (Pironon et al., 2017; Pujol & Pannell, 2008). On one hand, “leading edge” populations at high latitudes may have higher genetic variation due to gene flow from pre‐adapted, lower latitude populations, whereas “trailing edge” populations at low latitudes probably lack gene flow from pre‐adapted populations (Davis & Shaw, 2001). On the other hand, populations at low‐latitude range limits may exhibit unique genetic variation due to their longer history and persistence in extreme environments (Hampe & Petit, 2005; Pironon et al., 2017).

By altering important environmental cues, climate change can impose selection on phenological traits that dictate the timing of key life cycle events (Inouye, 2008). As a result, many taxa have exhibited phenological shifts in response to recent climate change (Root et al., 2003; Walther et al., 2002), likely through a combination of plastic and evolutionary changes. Flowering time is a key phenological trait in angiosperms, as flowering too early risks experiencing a late frost which can be detrimental to fruit production (Ågren, 1988). Conversely, flowering too late may reduce seed count and prevent reproduction before the onset of winter (Totland, 1997). Flowering time has advanced with warmer temperatures (Petrauski et al., 2019) and drought (Franks et al., 2007). Under climate change, longer growing seasons may select for later flowering to allow more time for growth (Weis et al., 2014). Alternatively, drought may truncate the growing season and select for earlier flowering (Franks et al., 2007). In contrast with drought avoidance (e.g., via increased water use efficiency) and drought tolerance (e.g., via increased root growth), advanced phenology is part of a drought escape strategy, whereby plants complete reproduction before the onset of drought (Kooyers, 2015). Populations can harbor high genetic variation for flowering time (Sheth & Angert, 2016). Thus, the evolution of phenological traits, which can be both highly heritable (Foolad & Jones, 1992; Weber & Moorthy, 1952) and under selection due to climate change (Dickman et al., 2019; Franks et al., 2007), may boost the growth rates of populations declining due to recent environmental change. Multiple studies have already found evidence for rapid evolutionary responses to recent climate change in flowering time (Anderson et al., 2012; Franks et al., 2007; Hamann et al., 2018; Thomann et al., 2015).

Evolutionary shifts in phenology may result in correlated evolutionary responses in other traits (Etterson & Shaw, 2001). Even if sufficient genetic variation is present for evolution to occur in a single trait, genetic correlations antagonistic to the direction of natural selection could constrain evolutionary responses (Etterson & Shaw, 2001). Earlier flowering has been associated with decreased vegetative growth (Colautti et al., 2010; Hall & Willis, 2006) due to reductions in the time available for vegetative growth prior to flowering. Under warmer temperatures, selection for greater vegetative growth due to the longer growing season could therefore be antagonistic to selection for earlier flowering due to drought. Understanding the traits correlated with flowering time and their potential trade‐offs is crucial to predicting evolutionary responses.

**FIGURE 1:**
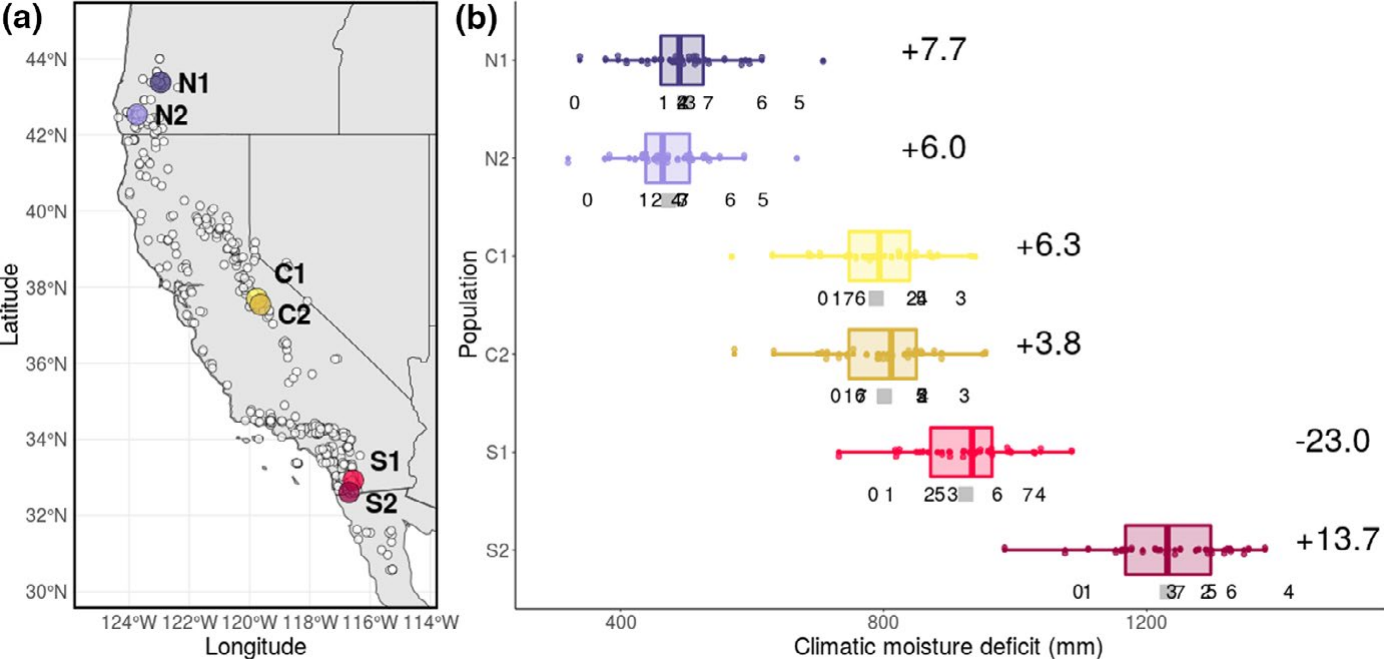
(a) Map of focal northern‐edge, central, and southern‐edge populations of*Mimulus cardinalis*(colored circles) and the locations of herbarium specimens (white circles, Angert et al., 2018). (b) climatic moisture deficit for each population from 2010 to 2017 (numbers below each box; 0:2010; 7:2017) with boxplots of historical data from 1980 to 2009. Gray squares indicate mean historical climatic moisture deficit for each population. Numbers to the right of each box indicate the average climatic moisture deficit anomaly (difference between contemporary and historical averages) for each population from 2010 to 2017, with positive anomalies representing recent increases and negative anomalies corresponding to recent decreases in climatic moisture deficit. We used the ClimateWNA v5.51 software package to obtain climate data for each population (available at ;http://tinyurl.com/ClimateNWA; Wang et al., 2016)

Here, we performed a resurrection study to examine evolutionary responses to recent climate change in populations across the geographic range of the scarlet monkeyflower, *Mimulus cardinalis*, a perennial herb that spans a broad latitudinal and climatic gradient in western North America (Figure 1a). *Mimulus cardinalis* occurs in a Mediterranean climate, where the greatest precipitation occurs in winter and soils continually dry as the growing season progresses (Muir & Angert, 2017). By growing ancestral and descendant seeds in a common environment, resurrection studies allow for the detection of evolutionary shifts in ecologically important traits (Dickman et al., 2019; Franks et al., 2007, 2017). We grew ancestors and descendants from northern‐edge, central, and southern‐edge populations of *M. cardinalis* before and after a period of severe drought and warming in western North America in a common environment to evaluate the following hypotheses. First, populations experiencing increased drought in recent years will evolve earlier flowering times. We predicted that if populations are adapting to recent drought, descendants should evolve earlier flowering time to escape the negative effects of drought (Kooyers, 2015). Climatic moisture deficit, an index of drought stress that integrates the effects of temperature and precipitation, has increased for all but one of our study populations, (Figure 1b; Wang et al., 2016) and drought often selects for earlier phenology (Franks et al., 2007; Hamann et al., 2018). Second, the magnitude of evolutionary response will vary among populations across the species' range. Information about standing genetic variation for flowering time suggests that evolutionary responses in phenology should increase from north to south. Specifically, the ancestral cohorts of southern‐edge populations harbored the greatest amount of genetic variation in flowering time, whereas northern‐edge populations harbored the lowest genetic variation (Sheth & Angert, 2016). Further, a southern‐edge population experienced the most extreme average increase in climatic moisture deficit over the study period (+13.7 mm in S2), whereas another experienced a mean decrease relative to historical conditions (−23.0 mm in S1, Figure 1b), suggesting that the strength of selection on flowering time could vary among populations. Third, populations that evolve earlier flowering will show correlated evolutionary responses in stem height. We predicted that individuals with earlier flowering times should have shorter stems at the time of flowering due to the shorter growth period prior to first flower.

## 2 MATERIALS AND METHODS

### 2.1 Study system and seed collections

*Mimulus cardinalis* (Phrymaceae), which typically flowers between May and September, is an herbaceous perennial that occurs below 2,400 m in wet habitats alongside streams from Baja California to Oregon in western North America (Figure 1a; Fraga, 2018; Lowry et al., 2019). *Mimulus cardinalis* has become a model system for studying local adaptation, geographic range limits, and responses to climate change (Angert, 2009; Angert & Schemske, 2005; Angert et al., 2011; Bayly & Angert, 2019; Paul et al., 2011; Wooliver et al., 2020). We chose *M. cardinalis* as the study system for this experiment due to the availability of seeds from phenotypically differentiated populations across the species' geographic range (Muir & Angert, 2017; Sheth & Angert, 2016). Previous work using the ancestral cohort of the same six populations showed greater genetic variation for flowering time at the southern range edge compared to the center and northern edge (Sheth & Angert, 2016). Demographic data from 2010 to 2014 suggest that three populations (N1, C2, and S1) have declined in recent years (Sheth & Angert, 2018), but such data are not available for the remaining populations.

We collected seeds from 57 to 216 individuals (representing unique maternal lines) in each of two northern‐edge, two central, and two southern‐edge populations in fall 2010 (ancestral cohort) and 2017 (descendant cohort, Table 1, Figure 1a, Table A1) as described in Sheth and Angert (2016). During this period, western North America experienced multiple years of record warming compounded by severe drought (Diffenbaugh et al., 2015; Griffin & Anchukaitis, 2014; Robeson, 2015; Wang et al., 2016; Figure 1b) that have likely imposed selection on traits involved in climate adaptation. Although not every season in every population was abnormally hot/dry, all populations experienced years of anomalous weather that likely imposed selection. Demographic surveys (2010–2014) found multiple years in which large fractions of the population died and/or no seedling recruitment occurred, leading to significant declines in 19 out of 32 populations spanning the latitudinal gradient (Sheth & Angert, 2018). For instance, in the southern most population, none of the plants measured in 2011 survived to 2012, and no new recruitment occurred (Sheth & Angert, 2018). Although *M. cardinalis* lives near streams, stream hydrology is driven by regional factors and these water sources can be ephemeral if winter precipitation is low. For example, in 2010 a southern population (S1; Figure 1a) occurred along a large creek; by 2017 the creek had completely dried out in some areas (Sheth, *pers. obs*.). Another southern population (S2; Figure 1a) inhabited a desert wash that was dry in both sampling years. In addition, the magnitude of change in climatic moisture deficit (relative to historical conditions) varied among the focal populations, which could give rise to differences in the strength of selection on traits involved in adaptation to drought across the latitudinal range (Figure 1b).

**TABLE 1:** Latitude, longitude, and elevation for each study population *of Mimulus cardinalis*, along with sample sizes (*N*) for flowering time for the 2010 ancestral and 2017 descendant cohorts

Population	Latitude (°)	Longitude (°)	Elevation (m)	*N*_2010_	*N*_2017_
N1	43.37876	−122.95207	295	46	156
N2	42.53529	−123.73016	914	88	169
C1	37.70377	−119.75363	1,316	160	192
C2	37.54576	−119.64152	1,228	52	67
S1	32.92788	−116.56019	1,252	193	150
S2	32.60831	−116.70098	252	188	103

**FIGURE 2:**
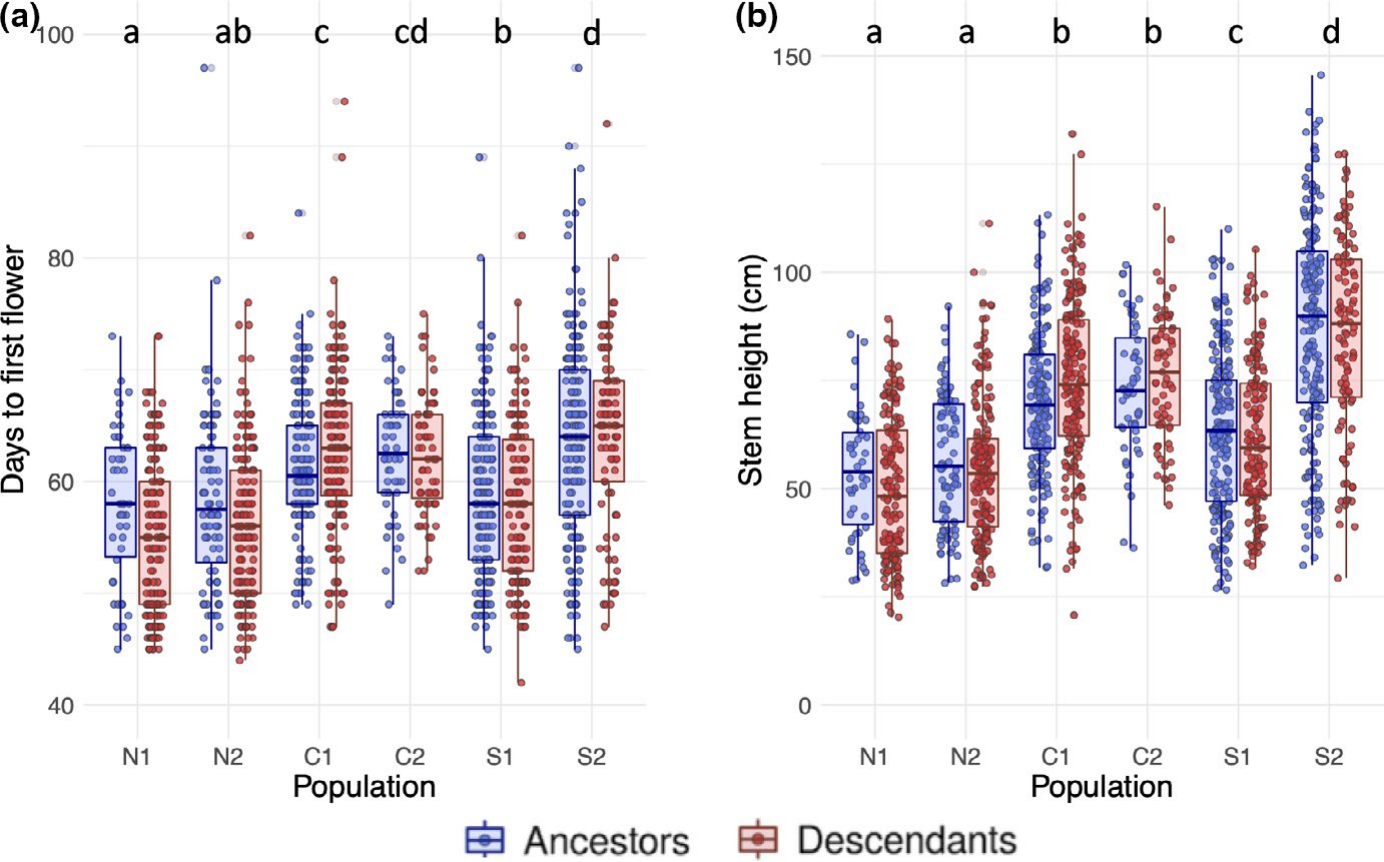
Flowering time, measured as number of days from germination to first flower (a), and stem height, measured in centimeters at day of first flower (b), for the 2010 ancestral and 2017 descendant cohorts of each population. Horizontal bars show median trait values, boxes show the inter‐quartile range, and whiskers correspond to the most extreme values within 1.5× the inter‐quartile range. There were no significant differences between ancestors and descendants within any population. Different letters indicate populations that were statistically different based on Tukey tests (*α* = 0.05). To ease interpretation, all graphical representations show untransformed data

### 2.2 Resurrection experiment

To evaluate evolutionary responses in flowering time and correlated responses in stem height across the geographic range of *M. cardinalis*, we performed a resurrection study in which plants from 2010 and 2017 were grown in the Fox Science Teaching Greenhouses at North Carolina State University. In May 2018, we filled 3‐inch pots with Jolly Gardner Pro‐Line C/P growing mix (Oldcastle Lawn and Garden Southeast, Pageland, South Carolina, USA) and planted three seeds from a given maternal family into each pot. Day/night temperatures were programmed to ~23°C/~18°C, with night temperatures implemented from 19:00 to 06:30. There was no supplemental lighting. To avoid accidentally moving seeds while watering, we carefully misted pots daily from arm's length above the trays. Families and cohorts within each population were randomized across trays to prevent competition among plants of different sizes. Initially, plants were configured in 56 trays with 32 pots per tray, in four rows by eight columns in each tray. The trays' locations were completely randomized across three benches, such that plants from every population and cohort were represented on each bench. We scored germination (emergence of cotyledons) of each seed daily for the first 4 weeks after planting and measured germination time as the number of days from planting to the emergence of cotyledons. After the majority of the seedlings had germinated and grown to a sufficient size (~4 weeks), we thinned seedlings to one randomly selected seedling (with a known germination date) per pot. We sub‐irrigated the plants daily. As the plants grew, we spread them out into 112 trays with 16 pots each, with a space between each pot. Approximately 5 weeks after planting and before plants started flowering, we began to fertilize the plants once a week using Peter's Excel 15‐5‐15 Cal‐Mag mix (Everris). Plants were treated as needed with fungicide and pesticide due to the presence of mold, fungus gnats, and shore flies.

We scored the date that each plant had its first open flower (when the corolla had opened with stamens and stigma visible) on a daily basis to measure flowering time as the number of days from germination to first open flower. We also measured the height (in cm) of the primary stem at first flower. Due to mortality and the breaking of primary stems prior to first flower, the final sample size for our analysis for flowering time and stem height ranged from 46 to 193 individuals per population and cohort combination, with a total sample size of 1,564 individuals (Table 1). The experiment concluded when the last plant flowered on August 26, 2018.

### 2.3 Seed mass and maternal effects

Three types of bias could potentially lead to erroneous conclusions of evolutionary responses in our resurrection study. First, the “invisible fraction” describes the phenotypes that are absent from the ancestral cohort due to nonrandom mortality during storage (Weis, 2018). Despite this potential bias, germination success was high in all populations and cohorts, indicative of minimal invisible fraction bias (Table A1). Second, storing ancestral seeds over multiple years can cause plastic responses to storage conditions and potentially cause differences between cohorts in adult traits such as flowering time (Franks et al., 2019). Third, ancestral and descendant seeds could have developed in distinct maternal environments in the field, potentially resulting in phenotypic differences between cohorts (Franks et al., 2019).

**FIGURE A1:**
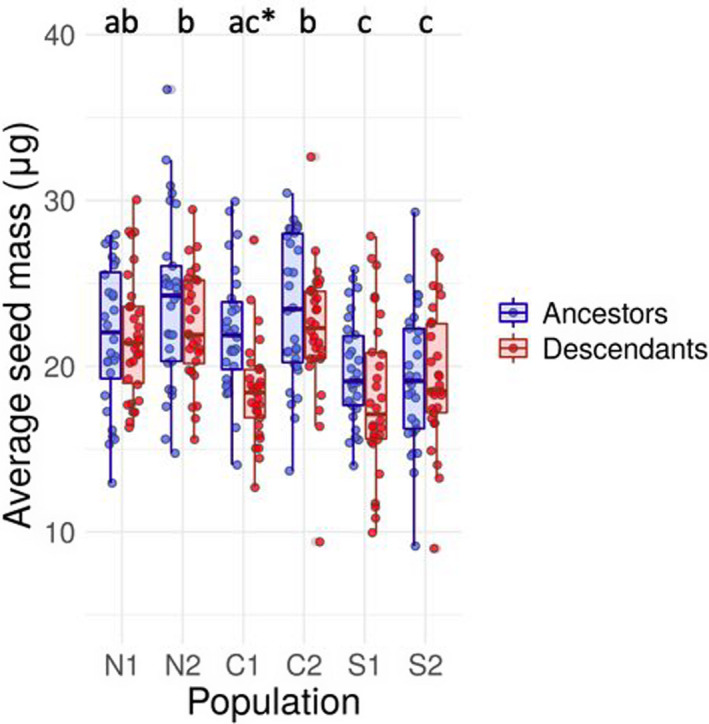
Seed mass (μg) for the 2010 ancestors and 2017 descendants of each population of*Mimulus cardinalis*. Horizontal bars show median seed mass, boxes show the interquartile range, and whiskers correspond to the most extreme values within 1.5× the interquartile range. Asterisks indicate a significant difference between ancestors and descendants within a given population, and different letters indicate populations that were statistically different based on Tukey tests (*α* = 0.05)

To account for maternal and storage condition effects, we weighed seeds for a subset of seed families. For each of 28–30 families per population and cohort, we weighed approximately 20 seeds using a microbalance. We calculated average seed mass for each family by dividing the mass in micrograms (μg) by the number of seeds weighed. We performed an ANOVA with seed mass as the response variable and population, cohort, and their interaction as explanatory variables. There was a statistically significant effect of population (*F*_5_ = 11.858, *p* < 0.001) and cohort (*F*_1_ = 9.493, *p* = 0.002), but the interaction between population and cohort was not significant (*F*_5_ = 1.591, *p* = 0.119). Seeds from 2010 were on average heavier than those from 2017 (absolute mean difference = 1.341 μg). Seed mass varied among populations, and generally decreased from north to south (Figure A1). In addition, we performed Spearman's tests for correlations between our focal traits and seed mass for each population, and corrected for multiple comparisons using the Bonferroni method. Despite the statistically significant effect of cohort on seed mass described above, seed mass was not correlated with germination time, flowering time or stem height in any of the populations (*p* > 0.05; Table A2). These results suggest that storage conditions and the maternal environment could have unknown effects on our evolutionary inferences, but because there were no statistically significant correlations between seed mass and our focal traits we did not include seed mass as a covariate in our models.

### 2.4 Statistical analysis

To test for evolutionary responses in flowering time and stem height, and whether the magnitude of evolutionary response varied across the species' range, we used linear models with each trait as the response variable and population, cohort, and their interaction as explanatory variables. To account for differences in the amount of natural sunlight that plants received, which was uneven across the three greenhouse benches, we included a bench effect and its interactions with other explanatory variables in models for all traits. We also included an effect of pot location and its interaction with other explanatory variables. Pot location describes whether the pot was at the center or edge of the tray, where pots in the center were more shaded by neighbors than those at the edge. We then compared these full models to nested models and used Akaike's Information Criterion (AIC) for model selection, with preference given to simpler models if AIC was less than 4 (Table A3, Burnham et al., 2011). The simplest nested model included population, cohort, and their interaction. We log‐transformed flowering time to improve normality. Although even log‐transformed flowering time deviated from the assumption of normality, linear models are robust to slight deviations, especially given a large sample size (Lumley et al., 2002). Additionally, nonparametric analyses such as the Kruskal–Wallis test could not accommodate interactions between explanatory variables. In the models for our focal traits, a statistically significant effect of population would mean that the trait varied among populations, an effect of cohort would indicate that the trait differed between ancestors and descendants (suggesting an evolutionary response), and a population‐by‐cohort interaction would reveal variation in evolutionary responses among populations. When there were statistically significant main effects, we used Tukey tests to determine which groups differed in mean trait values.

We excluded plants with unreliable data due to broken primary stems and irregular growth patterns from crowding. In addition to linear models, we performed Spearman's correlation tests between flowering time and stem height for each population to test for trade‐offs between growth and reproduction, and corrected for multiple comparisons using the Bonferroni method. For all analyses, we evaluated statistical significance at *α* = 0.05. We performed all statistical analyses in R statistical software, version 3.5.3 (R Core Team, 2019).

## 3 RESULTS

### 3.1 Flowering time

The final model for flowering time included population, cohort, bench, and the population‐by‐cohort interaction as explanatory variables. Population (*F*_5_ = 56.457, *p* < 0.001), bench (*F*_2_ = 17.786, *p* < 0.001), and the interaction between population and cohort (*F*_5_ = 2.784, *p* = 0.0164) had significant effects on flowering time, but cohort did not (*F*_1_ = 1.816, *p* = 0.178). There were no statistically significant differences between any population‐cohort combinations of interest, indicating that flowering time did not evolve between 2010 and 2017 in any population (Figure 2a). Flowering time differed more between regions (i.e., N, C, and S) than within regions (i.e., N1 vs. N2). N1 and N2 did not significantly differ in flowering time; nor did C1 and C2, although the S2 population flowered later than S1 (Figure 2a). N1 and N2 flowered earlier than both central populations and the S2 population, but N2 was not different from S1 (Figure 2a). C1 and C2 flowered later than S1 but C2 was not different from S2, while C1 flowered significantly earlier than S2 (Figure 2a). There was a statistically significant effect of bench on flowering time (*F*_2_ = 17.786, *p* < 0.001), such that plants on bench 1 (which received less sunlight than the other benches) flowered later than plants on benches 2 (absolute mean difference = 2.623 d) and 3 (absolute mean difference = 2.653 d). However, our results did not change when we repeated the analysis after omitting plants from bench 1.

### 3.2 Stem height

**FIGURE 3:**
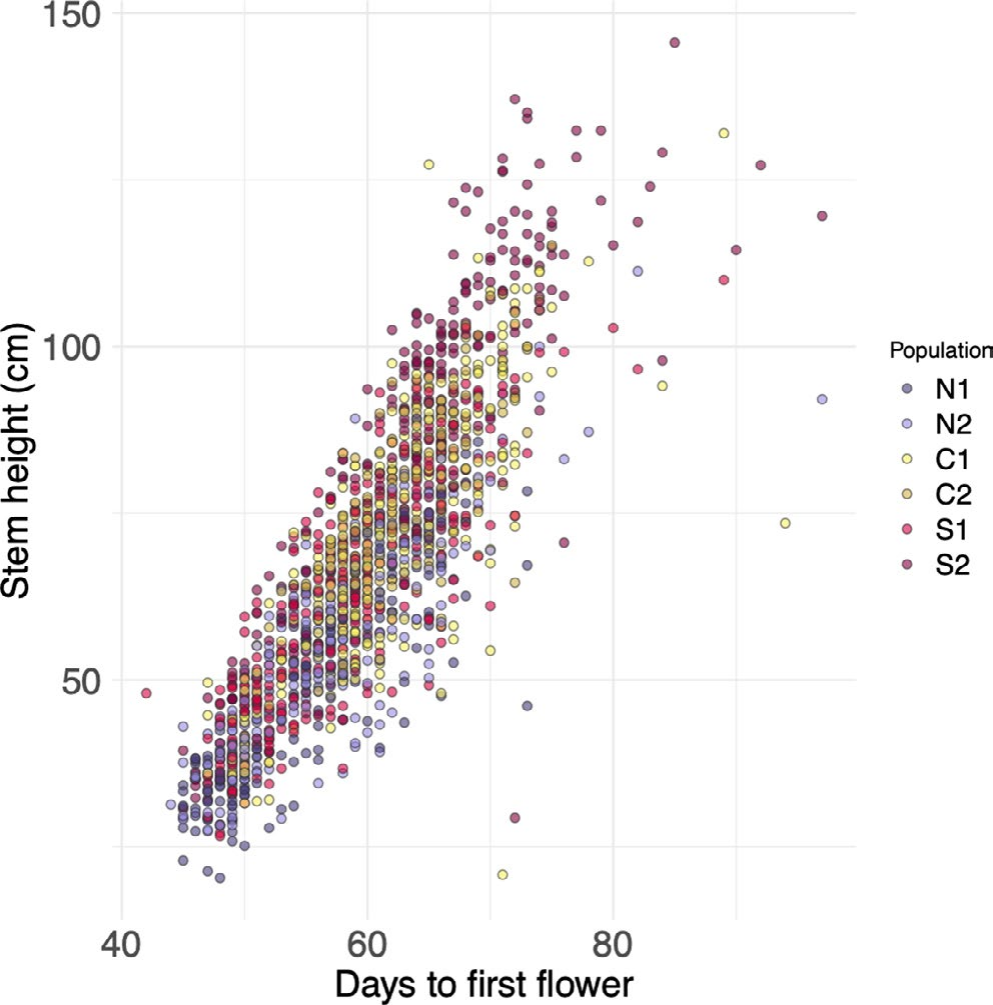
Relationship between flowering time and primary stem height at first flower for six focal populations of*Mimulus cardinalis*. Stem height was positively correlated with days to first flower for all populations (Table A4). Untransformed data are shown to ease interpretation

The final model for stem height at first flower included population, cohort, bench, pot location, and the population‐by‐cohort interaction as explanatory variables. Population (*F*_5_ = 137.917, *p* < 0.001), bench (*F*_2_ = 22.314, *p* < 0.001), and pot location (*F*_1_ = 40.565, *p* < 0.001) had significant effects on primary stem height, while cohort (*F*_1_ = 0.016, *p* = 0.898) and the population‐by‐cohort interaction (*F*_5_ = 1.955, *p* = 0.083) did not. Stem height significantly differed among all population pairs except for C1 versus C2 and N1 versus N2 (Figure 2b). With the exception of S1, stem height increased from north to south (Figure 2b). Plants on bench 1 were significantly taller at first flower than those on benches 2 (absolute mean difference = 9.668 cm) and 3 (absolute mean difference = 7.529 cm). Plants in the center of trays were larger at first flower than those at the edge of trays (absolute mean difference = 6.868 cm). There was a positive correlation between stem height and flowering time in all populations (Figure 3; Table A4).

## 4 DISCUSSION

Here, we performed a resurrection study to assess the evolution of phenology in response to a period of record drought and warming across a species' geographic range. As drought intensifies, the advancement of phenology may be advantageous by allowing populations to escape harsh conditions. Accordingly, we hypothesized that post‐drought descendants would evolve earlier flowering times relative to their pre‐drought ancestors, particularly in populations that have experienced increased drought in recent years (Figure 1b) and harbor sufficient genetic variation for phenology (Sheth & Angert, 2016). Contrary to our hypotheses, flowering time did not advance between ancestral and descendant cohorts in any population. Below, we discuss how seed storage conditions and maternal effects could have impacted our results. Given the measures we took to account for these biases, our findings still have important implications for studying rapid evolution in response to climate change. These findings suggest that the evolution of flowering time associated with drought escape is unlikely to rescue declining *M. cardinalis* populations, but mechanisms other than the evolution of drought escape may allow this species to persist with changing climate. Additionally, we predicted that populations that evolved earlier flowering would exhibit a correlated decrease in stem height because less time is available for vegetative growth prior to flowering. Although selection imposed by climate change may favor plants that flower early at a large size, our results point to a potential constraint on the evolution of early flowering and large size at flowering. Below, we discuss how the magnitude of climate change and genetic variation could explain the temporal trends we reported, and we interpret differences in focal traits across the species' range.

### 4.1 Evolutionary responses in flowering time and stem height

The probability of evolutionary rescue depends on the rate of environmental change and the amount of standing genetic variation present (Carlson et al., 2014). Given a strong selection event like drought and sufficient genetic variation for flowering time, we predicted the evolution of earlier flowering time if populations have adapted to recent climate change. Failing to support this hypothesis, flowering time did not evolve in any of the populations we examined. In an artificial selection experiment using the same populations, the southern populations had the greatest genetic variation and responses to selection on flowering time relative to central and northern populations (Sheth & Angert, 2016). We thus predicted that the southern populations would show the greatest advancement of flowering time. Contrary to this prediction, southern populations did not evolve earlier flowering times, and the northern populations showed the greatest advancement of flowering time from ancestors to descendants, though this result was not statistically significant. These northern populations, given additional time for evolutionary change, may exhibit more pronounced advancement over additional study years.

Selection for earlier flowering is often greater in higher latitudes, where shorter growing seasons necessitate earlier flowering so that plants can complete their life cycle before frosts (Munguía‐Rosas et al., 2011). Despite all but one study population experiencing greater climatic moisture deficit during the study years relative to historical conditions (Figure 1b), flowering time did not advance. Future studies measuring selection across space and time will improve our understanding of variation in evolutionary responses in phenology, and hence the likelihood of evolutionary rescue. At present, there is not published data on selection on flowering time across the latitudinal range of *M. cardinalis,* but such information would provide useful context for studies of rapid evolution in this species. Angert et al. (2008) documented selection for early flowering in advanced‐generation hybrids of *M. cardinalis* and *Mimulus lewisii* at high elevation. If selection at high elevation mirrors that at poleward latitudes, selection could also favor early flowering in poleward populations, but how selection acts on flowering time in this species at equatorial latitudes remains unknown. Although variation in climatic moisture deficit anomalies among populations indicates that selection on flowering time could differ across the species range, our focal populations showed similar evolutionary responses. These results suggest that rapid evolutionary shifts in flowering time associated with drought escape are unlikely to buffer *M. cardinalis* populations from recent climate change. However, *M. cardinalis* populations may persist via plastic shifts in phenology, the evolution of drought tolerance, and/or movement to track climatically suitable habitat. There is evidence of plasticity in flowering time in *M. cardinalis* in response to inter‐annual variation in the greenhouse environment (Sheth & Angert, 2016 *)*. Yet, plasticity in vegetative traits of *M. cardinalis* did not vary with latitude (Muir & Angert, 2017), which suggests that populations may rely more on adaptive evolution to respond to environmental change. Future manipulations of watering regimes would enable further assessments of phenological plasticity in this species.

Correlated traits antagonistic to the direction of selection could constrain the evolution of flowering time. Aside from phenology, we also examined how stem height varied across the species' range and between cohorts. Although stem height differed between the three regions and generally decreased with latitude (Figure 2), there was not an evolutionary response in stem height from ancestors to descendants. The effect of pot location, whereby plants in the center of trays were larger at first flower, is due to shading from surrounding neighbors. The positive correlation between stem height and flowering time in all populations and cohorts indicates that the earlier plants flower, the shorter their stems. This relationship could constrain evolutionary responses in phenology if selection favors both early flowering and a large size at flowering. Multiple studies have documented a genetic correlation between size at reproduction and flowering time (Colautti et al., 2010; Mitchell‐Olds, 1996). Drought might select for earlier flowering, but earlier flowering plants are smaller due to this phenotypic correlation and may therefore have lower reproductive output. Future work that examines multivariate constraints on adaptation to climate change is needed.

Although flowering time did not evolve to be earlier in response to drought, it varied among populations across the species' geographic range. Compared to S2 the S1 plants flowered earlier and were smaller at flowering, likely due to the higher elevation of the S1 site (Table 1). Additionally, flowering time demonstrated a latitudinal cline across the six study populations and tended to increase from north to south. This latitudinal cline indicates genetic differentiation in flowering time across the range, which may result in future differences in adaptive response and persistence. Previous work on *M. cardinalis* found a stronger latitudinal cline than that found in this study, which may be due to genotype‐by‐environment interactions caused by differences in growing conditions between studies (Sheth & Angert, 2016). Due to the positive correlation between flowering time and stem height, stem height also generally increased from north to south, with the exception of the S1 population.

### 4.2 Caveats

One major caveat of our study is the lack of a refresher generation, which could result in invisible fraction bias and storage condition effects. Invisible fraction bias is most worrisome when seed survival is low and nonrandom (Franks et al., 2019; Weis, 2018). However, germination success was similar across cohorts (Table A1), with all population‐cohort combinations having over 80% germination success when measured as % of families that germinated (Table A1). This high germination success across all populations and cohorts provides little evidence for high, nonrandom mortality in the ancestral cohort and thus negligible invisible fraction effects. Total germination success measured as % seeds that germinated in each population‐cohort combination ranged from ~68%–92% (Table A1), but success was not consistently lower in ancestors than descendants. However, the invisible fraction could have impacted the phenotypes observed in certain populations, potentially masking our ability to infer evolutionary changes between ancestors and descendants. In addition, artificially aging seeds to mimic storage condition effects has led to later flowering times compared to unaged seeds (Franks et al., 2019). Seeds that survive aging tend to be smaller, and smaller seeds may flower later due to lower provisioning, creating an invisible fraction bias due to the loss of earlier flowering individuals (Franks et al., 2019). If storage conditions had strongly impacted flowering time in the ancestral cohort, we might have inferred evolution of earlier flowering in descendants as an artifact rather than due to adaptation to recent climate change. Given that ancestors did not flower later than descendants in any population, storage condition effects on flowering time were likely minimal.

There are three additional caveats that limit our inferences of evolutionary responses in this study. First, rather than including a refresher generation to produce ancestral and descendant seeds in a common environment, we used field‐collected seeds, which could introduce maternal effects. Seed mass was not correlated with any of our focal traits, which indicates that maternal effects on these traits were negligible (Table A2). However, we acknowledge that since seed mass does not fully account for maternal effects, these effects could have still influenced our evolutionary inferences. Second, *M. cardinalis* is a perennial herb, and 7 years may be an insufficient amount of time for the rapid evolution of traits. However, a recent demographic study showed low survival in northern and southern populations (Sheth & Angert, 2018), suggesting shorter generation times and thus greater potential for evolutionary responses in these populations relative to central populations. Moreover, selective mass mortality events associated with climate change can result in evolutionary responses even in longer‐lived species (Nadeau & Urban, 2019). Future studies that examine this species over a greater time period may find more evidence for the evolution of drought escape. Finally, a previous study in the central part of the species' range showed that a small fraction of *M. cardinalis* seeds can persist in the seed bank for a year or more (Angert, 2005). The persistence of seeds in the seed bank suggests that the seeds collected for this study could have been from a past year and could potentially dampen evolutionary responses (Dickman et al., 2019; Franks et al., 2007).

### 4.3 Conclusions

In the context of climate change, the rapid evolution of earlier phenology may allow some species to escape stressful drought conditions (Franks et al., 2007; Hamann et al., 2018). None of the populations in this study have evolved earlier phenology in response to climate change, which could indicate that they are unable to cope with the long‐term effects of drought, that the drought was not as strong of a selective event as we assumed, that insufficient time has passed for evolutionary change, or that genetic correlations are constraining evolution. At least three of the study populations are already facing declines due to climate change (Sheth & Angert, 2018), but showed limited evolution in phenology over the studied timeframe. These findings suggest that these populations may need to rely on range shifts, phenotypic plasticity, or the evolution of other ecological traits to cope with climate change (Wooliver et al., 2020). We caution that maternal effects, seed storage conditions, and the invisible fraction could have masked our inferences of evolutionary responses. Nonetheless, the absence of a correlation between seed mass and our focal traits, along with the uniformity of high germination success between cohorts suggests that these influences may have been minimal. Future studies should compare how evolutionary responses in both drought avoidance and escape traits vary across the species' range. Studies examining the contemporary evolution of traits involved in adaptation to climate change are necessary for forecasting population persistence and species' geographic distributions, but not all populations or species will be capable of rapid evolution in the face of increasing drought.

## CONFLICT OF INTEREST

The authors do not have any conflicts of interest.

## AUTHOR CONTRIBUTIONS

**Emma E. Vtipil:** Data curation (lead); formal analysis (lead); funding acquisition (supporting); investigation (lead); visualization (lead); writing – original draft (lead); writing – review and editing (equal). **Seema Nayan Sheth:** Conceptualization (lead); data curation (supporting); formal analysis (supporting); funding acquisition (lead); investigation (supporting); methodology (lead); project administration (lead); supervision (lead); visualization (supporting); writing – original draft (supporting); writing – review and editing (equal).

## Data Availability

All data and scripts associated with this manuscript are available at https://doi.org/10.5061/dryad.bvq83bk72.

## 1

**TABLE A1:** Germination success for ancestral (2010) and descendant (2017) cohorts of each population

Population	*N*_seeds planted_	*N*_seeds germinated_	Percent of seeds germinated	*N*_family planted_	*N*_family germinated_	Percent of families germinated
N1 2010	171	121	70.8	57	48	84.2
N1 2017	489	434	88.8	163	158	96.9
N2 2010	309	235	76.1	103	92	89.3
N2 2017	528	487	92.2	176	176	100
C1 2010	612	418	68.3	204	168	82.4
C1 2017	600	527	87.8	200	196	98.0
C2 2010	174	152	87.4	58	54	93.1
C2 2017	243	180	74.1	81	67	82.7
S1 2010	648	547	84.4	216	195	90.3
S1 2017	558	395	70.8	186	153	82.3
S2 2010	618	535	86.6	206	195	94.7
S2 2017	345	282	81.7	115	107	93.0

Note

*N*_seeds planted_: the number of seeds planted; *N*_seeds germinated_: the number of seeds germinated; *N*_family planted_: the number of families planted; *N*_family germinated_: the number of families germinated.

We calculated the percent of seeds that germinated (*N*_seeds germinated_/*N*_seeds planted_), and the percent of families that germinated (*N*_family germinated_/*N*_family planted_).

**TABLE A2:** Correlation between average seed mass and focal traits

Population	Germination time *ρ* (*P)*	Flowering time *ρ* (*P*)	Stem height *ρ* (*P*)
N1	0.022 (1.00)	−0.080 (1.00)	−0.149 (1.00)
N2	−0.073 (1.00)	0.016 (1.00)	0.113 (1.00)
C1	−0.340 (0.058)	−0.093 (1.00)	−0.169 (1.00)
C2	−0.217 (0.574)	−0.050 (1.00)	−0.046 (1.00)
S1	−0.213 (0.612)	0.152 (1.00)	0.140 (1.00)
S2	−0.163 (1.00)	−0.083 (1.00)	−0.070 (1.00)

Note

Spearman's rank correlation *ρ* and associated *p*‐value for the relationship between seed mass (μg) and germination time (days), flowering time (days), and stem height (cm) within each population. *p*‐values are corrected for multiple comparisons via the Bonferroni method (*α* = 0.05).

**TABLE A3:** Akaike's Information Criterion (AICc) for full and nested models of flowering time and stem height

Response variable	Model	AICc
Flowering time	P + C + B + L + P * C + P * B + P * L + C * B + C * L + B * L + P * C * B + P * C * L + P * B * L + C * B * L + P * C * B * L	−2,157.9
	P + C + B + L + P * C + P * B + P * L + C * B + C * L + B * L + P * C * B + P * C * L + P * B * L + C * B * L	−2,169.1
	P + C + B + L + P * C + P * B + P * L + C * B + C * L + B * L + P * C * B + P * C * L + P * B * L	−2,163.6
	P + C + B + L + P * C + P * B + P * L + C * B + C * L + B * L + P * C * B + P * C * L	−2,180.3
	P + C + B + L + P * C + P * B + P * L + C * B + C * L + B * L + P * C * B	−2,187.2
	P + C + B + L + P * C + P * B + P * L + C * B + C * L + B * L	−2,203.1
	P + C + B + L + P * C + P * B + P * L + C * B + C * L	−2,204.5
	P + C + B + L + P * C + P * B + P * L + C * B	−2,206.4
	P + C + B + L + P * C + P * B + P * L	−2,204.8
	P + C + B + L + P * C + P * B	−2,209.1
	P + C + B + L + P * C + P * L	−2,216.7
	P + C + B + L + P * C + C * B	−2,222.2
	P + C + B + L + P * C + C * L	−2,218.9
	P + C + B + L + P * C	−2,220.9
	**P** + **C** + **B** + **P * C**	**−2,220.1**
	P + C + P * C	−2,189.7
Stem height	P + C + B + L + P * C + P * B + P * L + C * B + C * L + B * L + P * C * B + P * C * L + P * B * L + C * B * L + P * C * B * L	13,567.8
	P + C + B + L + P * C + P * B + P * L + C * B + C * L + B * L + P * C * B + P * C * L + P * B * L + C * B * L	13,550.1
	P + C + B + L + P * C + P * B + P * L + C * B + C * L + B * L + P * C * B + P * C * L + P * B * L	13,551.8
	P + C + B + L + P * C + P * B + P * L + C * B + C * L + B * L + P * C * B + P * C * L	13,535.0
	P + C + B + L + P * C + P * B + P * L + C * B + C * L + B * L + P * C * B	13,527.1
	P + C + B + L + P * C + P * B + P * L + C * B + C * L + B * L	13,513.0
	P + C + B + L + P * C + P * B + P * L + C * B + C * L	13,514.3
	P + C + B + L + P * C + P * B + P * L + C * B	13,513.5
	P + C + B + L + P * C + P * B + P * L	13,513.5
	P + C + B + L + P * C + P * B	13,505.2
	P + C + B + L + P * C + P * L	13,503.2
	P + C + B + L + P * C + C * B	13,495.2
	P + C + B + L + P * C + C * L	13,496.4
	**P** + **C** + **B** + **L** + **P * C**	**13,495.1**
	P + C + B + P * C	13,534.2
	P + C + P * C	13,571.4

Note

P: population; C: cohort; B: bench; L: pot location.

For flowering time and stem height, the most complex model included population, cohort, bench, pot location, and their interactions. For all traits, the simplest model included population, cohort, and their interaction. When ΔAIC was <4, we chose the simplest model. Final models are shown in bold.

**TABLE A4:** Spearman's rank correlation *ρ* for the relationship between primary stem height and flowering time in each population

Population	*ρ*
N1	0.870
N2	0.838
C1	0.780
C2	0.762
S1	0.859
S2	0.873

Note

All correlation coefficients were statistically significant after applying Bonferroni correction for multiple tests (*α* = 0.05).
